# Editorial: Methods in applied genetic epidemiology 2022

**DOI:** 10.3389/fgene.2024.1469497

**Published:** 2024-09-05

**Authors:** Qi Guo, Christopher Neal Foley, Zhana Kuncheva

**Affiliations:** ^1^ Insmed Innovation UK, Cambridge, United Kingdom; ^2^ bioXcelerate, Optima Partners Ltd, Edinburgh, United Kingdom; ^3^ Bayes Centre, The University of Edinburgh, Edinburgh, United Kingdom

**Keywords:** genetic epidemiology, human genetics, rare genetic variant analysis, gene-environment interaction, genetic testing, diverse populations

Genetic Epidemiology has unprecedented potential to further our understanding of human disease aetiology, progression, and prevention. The development of genetics and genomics methods and tools has transformed the way in which scientists discover and interpret the genotype-phenotype association, enabling better understanding of human disease biology. Despite the huge effort made in this field, there is still much work remaining to be done, such as enhancing the study of human genetics and genomics in diverse populations, rare genetic variant analysis and gene-environment interaction.

The Research Topic “Methods in Applied Genetic Epidemiology” showcases new developments and recent advances in experimental techniques and analysis methodologies in the field with applications to diverse populations covering different ethnic and demographic groups. This Research Topic comprises one method article, which compares the performances of different longitudinal methods in the context of modelling polygenic risk for post-traumatic stress disorder over time in individuals of African and European ancestry, and four original research articles ranging from rare variant association analysis, gene by gene/environment interaction to identify rare Thalassemia variants using multiple genetic methods.

Genome-wide association studies (GWAS) have identified thousands of genetic loci associated with a myriad of diseases, offering critical insights into the biological pathways involved. However, the field is moving beyond single-marker analyses towards more integrative approaches and gene set-based associations. Polygenic risk scores (PRS) represent a significant leap, allowing for the aggregation of multiple genetic variants to predict individual risk profiles more accurately. Passero et al. investigate various longitudinal modelling approaches to determine how these methods compare when analysing trajectory changes of a continuous, repeatedly measured phenotype. The authors evaluate the power, false positive rate, estimation accuracy, and precision of five models alongside naïve linear regression with a simulation study. By incorporating PRS in the longitudinal model, they study post-traumatic stress disorder over time in individuals of African and European ancestry. The advent of whole-genome sequencing and whole-exome sequencing has unveiled the intricate landscape of rare genetic variants. These technologies enable researchers to explore the full spectrum of genetic variation. On the rare variant association space, Falk et al. extend the convex-optimised sequence kernel association test for rare variant studies that learns from data the optimal convex combination of kernels, to the full Generalised Linear Model setting with arbitrary non-genetic covariates. They show that the resulting optimisation problem is a quadratic programming problem that can be solved with no additional cost. A modified objective is related to an upper bound for the *p*-value through a decreasing exponential term in the objective function, indicating that optimising this objective function is a principled way of learning the combination of kernels. The proposed new method enables correcting for important confounders in association studies such as age, sex or population structure for both quantitative and binary traits. The authors show that this new approach can recover sensible weights and achieve higher power across different sample sizes and misspecification settings.

Genetic epidemiology also seeks to unravel the complex interplay between genetic variations and environmental factors in the context of human health and disease. Gene and environment interactions are often cited as a potential source for the “missing heritability”. Recent advances in multi-omics data technology have revolutionised genetic epidemiology research. High-throughput technologies now allow comprehensive analysis of genomes, epigenomes, and transcriptomes at unprecedented scale and resolution. The integration of these multi-omic data sets provides a holistic view of biological processes, enabling researchers to uncover complex gene-environment interactions and regulatory mechanisms underlying disease susceptibility. These advancements hold immense potential for elucidating the genetic architecture of complex diseases and improving health outcomes globally. Majarian et al. conduct a study to examine whether lifestyle exposures can alter the association between genetic variants and cardiometabolic outcomes in diverse populations. They leverage publicly available genetic, epigenomic and transcriptomic summary association data to identify a subset of interactions among which multiple forms of evidence point toward potential mechanistic explanations. They also observed an overrepresentation of prioritised interaction loci drawn from meta-analyses of African-ancestry populations. Liu et al. report a study aiming to detect the associations of SNPs in a Research Topic of genes with Cardiovascular disease and Ischemic Stroke as well as gene and gene/environment interactions in Chinese Han population. They explore the possible pathogenic mechanisms of these genes and provide a theoretical basis for early Cardiovascular disease and Ischemic Stroke prevention and treatment.

A significant breakthrough in the field is the integration of genetic testing in both research and clinical settings. Genetic testing has become a vital tool in identifying hereditary conditions and assessing individual disease risk. By combining genetic testing data with epidemiological methods, researchers can more precisely determine the genetic underpinnings of various conditions and tailor preventive and therapeutic strategies. Jiang et al. use multiple genetic methods to identify rare thalassemia variants for prenatal diagnosis. It is important to identify the genotypes of parents before prenatal thalassemia diagnosis, especially in cases with phenotypes not matching genotypes. They conclude that accurate gene diagnosis for probands with combined molecular biology techniques is the key to prenatal diagnosis of rare thalassemia. Multiple techniques for α globin cluster should be performed on cases with severe β−thalassemia who only carry a mutation in the HBB gene. Compared to other methods, single-molecule real-time sequencing offers advantages to find complicated genotypes of thalassemia.

The field of genetic epidemiology is rapidly advancing, driven by technological innovations and increased data availability. The small Research Topic of articles showcased in this Research Topic not only present several innovative techniques and methodology advancement but also highlight the importance of increasing the representation of diverse ethnic and demographic populations in genetic studies.

